# Warm Bath in Dropsy after Scarlet Fever

**Published:** 1848-04

**Authors:** 


*Warm Bath in Dropsy after Scarlet Feverr.*—Dr. Golding Bird emarks, that as a prophylactic remedy the warm bath is invaluable n the treatment of dropsy after scarlet fever. He scarcely recollects a case occurring, where the warm bath was daily used, as soon as the skin began to exfoliate, and continued until a healthy perspiring surface was obtained. I have for many years employed warm baths as here recommended; but several cases of dropsy having occurred under the treatment, I found that it happened in cases where the bath failed to produce *speedy* exfoliation of the skin. I then used, and for some years have continued to employ, a modification of the warm bath, and with uniform success. I direct a quantity of bran to be scalded, the bath then reduced to a proper temperature, and continued friction to be kept up with handfuls of the bran during the immersion. The skin is speedily removed.—*Provincial Med. and Surg. Jour.*

